# Neurobrucellosis in systemic lupus erythematosus 

**DOI:** 10.22088/cjim.8.2.119

**Published:** 2017

**Authors:** Jamshid Vafaeimanesh, Alireza Shahamzeh, Mohammad Bagherzadeh

**Affiliations:** 1 Gastroenterology and Hepatology Disease Research Center, Qom University of Medical Sciences, Qom, Iran.; 2 Gastrointestinal and liver Diseases Research Center, Iran University of Medical Sciences, Tehran, Iran.; 3 Qom University of Medical Sciences, Qom, Iran.; 4 Clinical Research Development Center, Qom University of Medical Sciences, Qom, Iran.

**Keywords:** Neurobrucellosis, Systemic lupus erythematosus

## Abstract

**Background:**

Brucellosis is a zoonotic infection which is endemic in many countries. It is a multisystem disease which may present with a broad spectrum of clinical manifestations and complications. Neurobrucellosis is an uncommon complication of brucellosis.

**Case presentation:**

A 25-year-old woman with a history of lupus for 5 months referred to the emergency ward of Shahid Beheshti Hospital of Qom due to vertigo, drop attack and a convulsion episode from the previous day. She was unable to move at initial evaluation, and her upper and lower extremities were spastic. She had blurred vision one day after admission. Based on her past history and suspecting neurological pulmonary presentations, treatment with immunosuppressive drugs was started and brain MRI was performed. According to the MRI mode and endemic area, neurobrucellosis was suspected and 2ME and Wright tests were performed. Wight test was 1.5120 while 2ME test was 1.640 which were strongly positive. So, with neurobrucellosis diagnosis, the patient was treated but unfortunately 4 days later, after respiratory apnea, she was pronounced dead.

**Conclusion:**

In endemic areas for brucellosis, neurobrucellosis should always be kept in mind in the differential diagnosis of neurological and psychiatric cases that are encountered.

Brucellosis is a zoonotic infection transmitted to humans by contact with fluids from infected animals (sheep, cattle, goats, pigs, or other animals) or derived food products such as unpasteurized milk and cheese. It is one of the most common zoonoses around the world (1). It has high morbidity both for humans and animals and is an important cause of economic loss and a public health problem in many developing countries (2). This systemic infection has a wide clinical spectrum, ranging from asymptomatic disease to severe and/or fatal illness (2). It’s clinical and laboratory features vary widely. Focal infection occurs in about 30% of cases and it can affect any organ system (2-4). Neurobrucellosis is an uncommon complication of brucellosis (5). Neurological involvement occurs in 0-7% of cases. Manifestations include meningitis (acute or chronic), encephalitis, myelitis, radiculitis, and/or neuritis (with involvement of cranial or peripheral nerves) (4, 6, 7). The mortality rate of neurobrucellosis in the postantibiotic era is 0-5.5% but permanent neurologic deficits, particularly deafness are common (8, 9). In this paper, we present a case of brucellosis with neurologic manifestations leading to death. 

## Case presentation

A 25-year-old woman referred to emergency ward of Shahid Beheshti Hospital of Qom due to vertigo, drop attack and one episode of convulsion from the previous day. At initial evaluation, she was unable to move and her upper and lower extremities were spastic. 

She had a lupus pneumonitis one month ago which was treated. The patient had a slight fever and initial laboratory tests revealed WBC 6800, Hb 11 mg/dL, PLT 188000, PT 15.2, INR 1.5 and PTT 37. She had blurred vision one day after admission. Based on the antecedents of the patient and suspecting neurological pulmonary presentations, immunosuppressive treatment with infusion of cyclophosphamide (1g Stat) and m*ethylprednisolone* (1g/day for 3 days) was initiated and brain MRI was performed. Lab tests showed ANA 2.9, Anti dsDNA 0.8, lupus anticoagulan Ab 42sec (Nl= 30-44), anticardiolipin IgG 6.3 IU/mL (NL <12), anticardiolipin IgM 4.6 IU/mL (NL <12), and anti beta-2 glycoprotein IgG 3 IU/mL (NL <10). Brain MRI showed disseminated white matter signal in periventricular area which extended to subcortical area and contained low T2 sequence which could be suggestive of infectious diseases. According to the MRI mode and endemic area, neurobrucellosis was suspected and 2ME and Wright tests were done. Wight was 1.5120 while 2ME was 1.640 which were strongly positive. A cerebrospinal fluid (CSF) sample was taken during the lumbopuncture (LP) disclosed Wright (1/160) with IgM 22.2 IU/mL (pos>22). So, the patient was treated with neurobrucellosis diagnosis, but unfortunately 4 days later, after respiratory apnea, she was pronounced dead.

**Figure 1:**
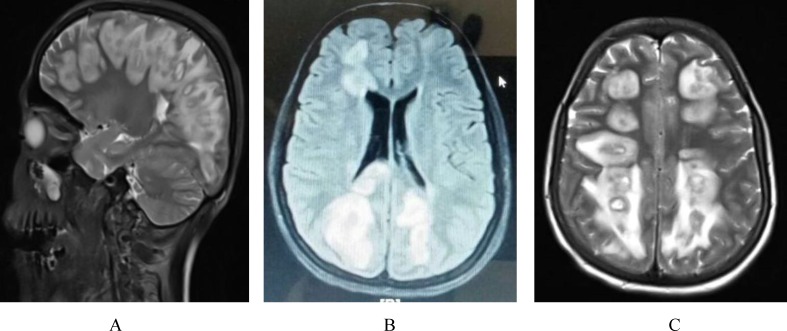
Sagittal T2W. Incidental note is made of maxillary sinusitis. (A), axial fluid-attenuated inversion recovery (FLAIR) Right aspect of corpus callusum (posterior part of body splenium junction) involvement (B), and axial T2W (C). Brain MRI images demonstrate the mild asymmetrical diffuse and dispread bilateral white matter abnormal high signal intensity at periventricular and centrum semiovale area

## Discussion

Brucellosis is endemic in many countries around the world. This multisystem disease may present with a broad spectrum of clinical manifestations and complications. CNS involvement of brucellosis is rare, but neurobrucellosis is an important complication. In a number of studies, the rate of neurobrocellosis has been reported <5% (6, 8, 10). In a referral center, this rate was 7.7% (11). In Iran, among 1996 cases of brucellosis, the incidence decreased from 17.1/100,000 in 2006 to 8.2/100,000 in 2009. In this study, the male: female ratio was 2:1 and the disease was most common in individuals aged 15-20 years (12). Neurobrucellosis may present in various forms but the most frequent clinical presentations are meningitis and  meningoencephalitis (8, 13). Also, headache, vomiting, fever and unconsciousness are common (14). Its neurological presentations include aphasia, dizziness, diplopia, hemiparesis, facial paralysis, tremor, and ataxia and its psychiatric symptoms incude depression, personality disorder, and halucinations. Meningeal irritation findings in neurobrucellosis have been reported at < 50% (15). These neurologic protests have been reported in some other systemic diseases. For example, neurologic and psychiatric symptoms were reported to occur in 10-80% of patients either prior to the diagnosis of Systemic Lupus Erythematosus (SLE) or during the course of their illness (16, 17). The wide range in reported prevalence reflects the use of different criteria for neuropsychiatric diseases. Different types of neurologic manifestations have been reported in patients with lupus which include central and peripheral manifestations. Central manifestations are aseptic meningitis, cerebrovascular disease, demyelinating syndrome, headache, movement disorder, seizure disorder, myelopathy, acute confusional state, anxiety disorder, cognitive dysfunction, and mood disorder. Psychosis and peripheral manifestations include Guillain-Barre syndrome, autonomic neuropathy, mononeuropathy, Myasthenia gravis, cranial neuropathy, plexopathy, and polyneuropathy (18). It is remarkable that some of these protests overlap in SLE and brucellosis and may cause diagnostic errors. In this patient, the primary diagnosis was neurologic manifestations of lupus and she was treated with immunosuppressive but neurobrucellosis should always be considered in the differential diagnosis of neurological and psychiatric cases that are encountered in endemic areas for brucellosis. Therefore, in patients whose clinical findings are compliant with neurobrocellosis, serological tests should always be performed both in serum and in CSF. The Coombs’ test is preferable as it is more sensitive compared to the SAT (Coombs 96.1%, SAT 88.2%) (19). Diagnosis of neurobrocellosis is usually made by the detection of specific antibodies in CSF and cultures of CSF are positive in less than one half of cases (20). 

In neurobrocellosis cases that are limited to CNS involvement, treatment with the combination of ceftriaxone, doxycyline and rifampicin is effective (5, 11). The mortality rate of neurobrucellosis in the postantibiotic era is 0-5.5% but permanent neurologic deficits, particularly deafness, are common (8, 9). Pedro-Pons et al. (6), in series of 42 neurobrucellosis cases, have reported only two cases of mortality while Haji-Abdolbagi et al. (21) reported one death of unidentified origin in a series of 31 cases. Akdeniz et al. (22) and Ceran et al. (5) did not report any deaths in their neurobrucellosis cases. 

In conclusion,** i**n endemic areas, neurobrucellosis should be considered in cases that have unusual neurological manifestations. Therefore, in patients whose clinical findings are compliant with neurobrucellosis, serological tests should always be performed both in serum and CSF.
